# De-escalation surgery for localized prostate cancer: rationale and future direction of precision prostatectomy

**DOI:** 10.1097/MOU.0000000000001434

**Published:** 2026-07-16

**Authors:** Johannes Franke, Craig G. Rogers, Laura Zuluaga, Ketan Badani, Mani Menon

**Affiliations:** a Department of Urology, Icahn School of Medicine at Mount Sinai, New York, New York, USA; b Department of Urology, Comprehensive Cancer Center, Medical University of Vienna, Vienna, Austria; c Vattikuti Urology Institute, Department of Urology, Henry Ford Health, Detroit, Michigan; d Mount Sinai Medical Center, Miami Beach, Florida, USA

**Keywords:** MPP, MPP version 2, precision prostatectomy, prostatectomy, seminal vesicle-sparing prostatectomy

## Abstract

**Purpose of review:**

The evolution of radical prostatectomy has resulted in excellent oncologic outcomes; however, postoperative erectile dysfunction remains a clinical constraint. While oncologic control in focal therapy remains its main limitation, Menon Precision Prostatectomy, also referred to as Millimeter Precision Prostatectomy (MPP) was developed to bridge this therapeutic gap. The purpose of this review is to summarize the rationale, development, and future direction of MPP.

**Recent findings:**

Advances in anatomical understanding have demonstrated that periprostatic innervation extends beyond the classical neurovascular bundles, with a complex network of autonomic fibers distributed along the prostatic capsule, Denonvilliers’ fascia, and the seminal vesicle complex. MPP was designed to enhance the preservation of these nerve-rich structures. In its initial iteration, a unilateral thin rim of prostatic tissue and the ipsilateral seminal vesicle were preserved, whereas dissection on the tumor-bearing side followed the principles of standard radical prostatectomy.

**Summary:**

Prospective studies demonstrated favorable oncologic outcomes with superior functional recovery compared to contemporary radical prostatectomy. However, the technically demanding subcapsular dissection raised concerns regarding generalizability. MPP Version 2 was developed as a modification of this concept. In this technique, the prostate is removed *en bloc*, while both seminal vesicles are preserved, thereby maintaining the integrity of their surrounding neural structures. This approach extends beyond prior seminal vesicle-sparing techniques, which were limited to partial preservation. MPP V2 is currently under clinical investigation, aiming to improve functional outcomes for patients undergoing prostatectomy.

## INTRODUCTION

Radical prostatectomy represents the gold standard for surgical management of prostate cancer. Robotic-assisted radical prostatectomy (RARP) provides excellent oncologic control in intermediate-risk patients, with prostate cancer-specific survival (CSS) exceeding 90% at 20 years; in favorable intermediate-risk patients achieving 95% CSS [1–6].

While robotic technology and nerve-sparing techniques have evolved, treatment-related erectile dysfunction remains common. Even in preoperatively potent men undergoing nerve-sparing RARP, 12-month erectile function recovery is far from guaranteed: published rates range widely from 23 to 90% [7,8^▪▪^]. This heterogeneity reflects age, baseline function, comorbidity, potency definition, and extent of nerve-sparing. Combined with excellent oncologic outcomes, it has shifted the discourse toward a risk-adapted de-radicalization of treatment, particularly in men with intermediate-risk disease, long life expectancy, and strong quality-of-life expectations.

While the development and increasing adoption of active surveillance and focal ablative techniques were developed to prevent functional morbidity, it has highlighted the competing risk of undertreatment, particularly in patients with intermediate-risk PCA, in whom residual disease and rates of secondary treatment remain elevated [9^▪^]. Functional outcomes of secondary salvage therapy after focal therapy may also be impaired when compared to modern-era upfront RARP cohorts [10].

To address this therapeutic gap, precision prostatectomy was developed, also referred to as Millimeter-, or Menon Precision Prostatectomy (MPP). MPP represents a risk-adapted surgical approach to enhance functional outcomes; the underlying rationale is the preservation of periprostatic tissue planes that harbor a substantial proportion of neural networks extending beyond the classical neurovascular bundle. 

**Box 1:**
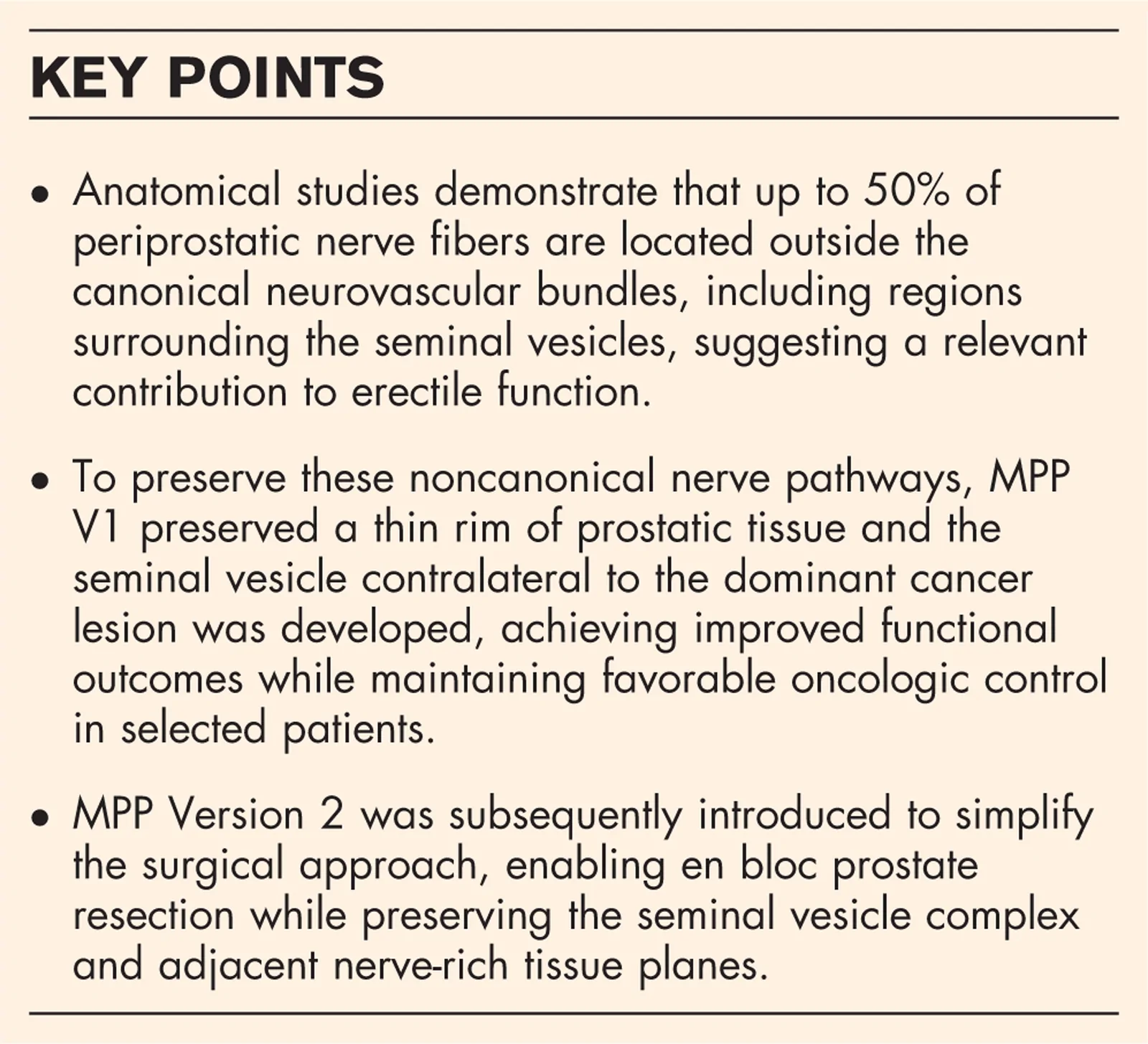
no caption available

## ANATOMICAL RATIONALE FOR PRECISION PROSTATECTOMY

While the classical model localizes cavernous nerves within the posterolateral neurovascular bundles, subsequent studies have demonstrated a far more complex periprostatic neural network. The roots of modern neural anatomy were first investigated by Finkle and Saunders [11]; later Walsh and Donker [12,13] identified the neurovascular bundle along the posterolateral aspects of the prostate and applied this to prostatectomy. The resulting nerve-sparing technique has significantly improved functional outcomes in prostatectomy. This canonical model of nerves traveling at 5 and 7 o’clock positions has guided surgical technique for decades; however, subsequent refined anatomical studies highlighted its limitations. Takenaka *et al.*[14] demonstrated in 2007, using intraoperative electrostimulation, that cavernosal nerves do not exclusively reside within the neurovascular bundle. Subsequent immunohistological staining studies by Sievert *et al.*[15] using PGP9.5 introduced the concept of neural periprostatic layer; studies by Ganzer *et al.* using S-100 and nitric-oxide synthase visualized that only 50% of NO-producing nerves reside inside the canonical bundles. They found a complex neural network distributed along the ventral capsule within Denonvilliers’ fascia and critically posterior to the SVs [16,17]. In-vivo examination using diffusion Tensor Magnetic Resonance Tractography in 2011 by Finley *et al.* visualized a lush network of fiber tracts around the prostate. With evolving technology, more granular micro-computed tomography analysis by Yang *et al.*[18] in 2021 has identified that nerves outside the neurovascular bundle run towards cavernosal structures. These noncanonical nerves would routinely be transected or torn during mobilization and removal of the seminal vesicle complex. Given their potential contribution to erectile function, their preservation provides a compelling anatomical rationale for de-escalating surgical treatment.

## CLINICAL RATIONALE FOR PRECISION PROSTATECTOMY

The functional significance of these anatomical findings is highlighted when supplemented with clinical observations of various pelvic surgical procedures. In patients undergoing prostate-sparing cystectomy [19] or simple prostatectomy [20], erectile function is often preserved despite extensive pelvic dissection. These approaches consistently preserve the prostatic capsule and the seminal vesicle region, where a substantial number of autonomic nerves reside. These observations are further supported by outcomes following nonnerve-sparing radical prostatectomy; in an MSKCC cohort, approximately 13% of patients regained erectile function despite deliberate bilateral transection of the canonical neurovascular bundles [21]. In contrast, when extending the perspective to visceral surgery, postoperative erectile dysfunction is frequently observed after lower anterior resection, even when the resection plane preserves the canonical neurovascular bundles [22]. In conclusion, these observations provide compelling clinical evidence that erectile function is not exclusively mediated within the classical model described by Walsh and Donker, and instead underscore the importance of preserving the integrity of nerve-rich tissue planes surrounding the prostate and seminal vesicle complex. Adapted nerve sparing was introduced by extending the tissue sparing to the veil of Aphrodite and the hood. By introducing modern grading of nerve sparing surgeons addressed the anatomical reality. Even though this led to improved functional outcomes, erectile dysfunction remains common [23,24,25^▪^].

Focal therapy was developed to address the functional limitations associated with radical treatment in selected patients with unifocal disease [26–29]; however, clinical experience has highlighted concerns regarding the consistency of oncologic control [30]. Under-detection of clinically significant multifocal disease may be a driver of oncologic failure. Pathological studies of prostatectomy specimens from patients who were deemed eligible for focal treatment, have demonstrated that lesion-directed therapy would have left behind clinically significant cancer in a substantial proportion of patients [31]. Even though primary functional outcomes after focal therapy appear compelling [32], data from systematic reviews suggest that erectile dysfunction is more common after salvage surgery when compared to similar cohorts of patients undergoing upfront radical prostatectomy [10].

To address the competing problems of oncologic control and functional morbidity, MPP was developed as a surgical solution for preserving nerve-rich planes while maximizing prostatic tissue extirpation.

## MPP VERSION 1: PATIENT SELECTION IN THE IDEAL FRAMEWORK

Precision prostatectomy was developed as a risk-adapted subtotal surgical approach for selected men with localized prostate cancer in whom preservation of erectile function was a major treatment priority. The concept was supported by an IDEAL stage 0 mapping study of radical prostatectomy specimens, which suggested that preserving a limited contralateral rim of prostatic tissue may reduce clinically significant residual disease compared with conventional focal ablation while maintaining a protective periprostatic tissue plane [33]. Inclusion criteria included PSA 20 ng/ml or less and clinical stage ≤cT2. A dominant unilateral cancer lesion had to be present, with no Gleason Pattern (GP) 4 on the contralateral side of the gland. In total, 88 patients were included in the IDEAL stage 1–2b prospective development studies, using these criteria [34].

## MPP VERSION 1: SURGICAL TECHNIQUE

**FIGURE 1:**
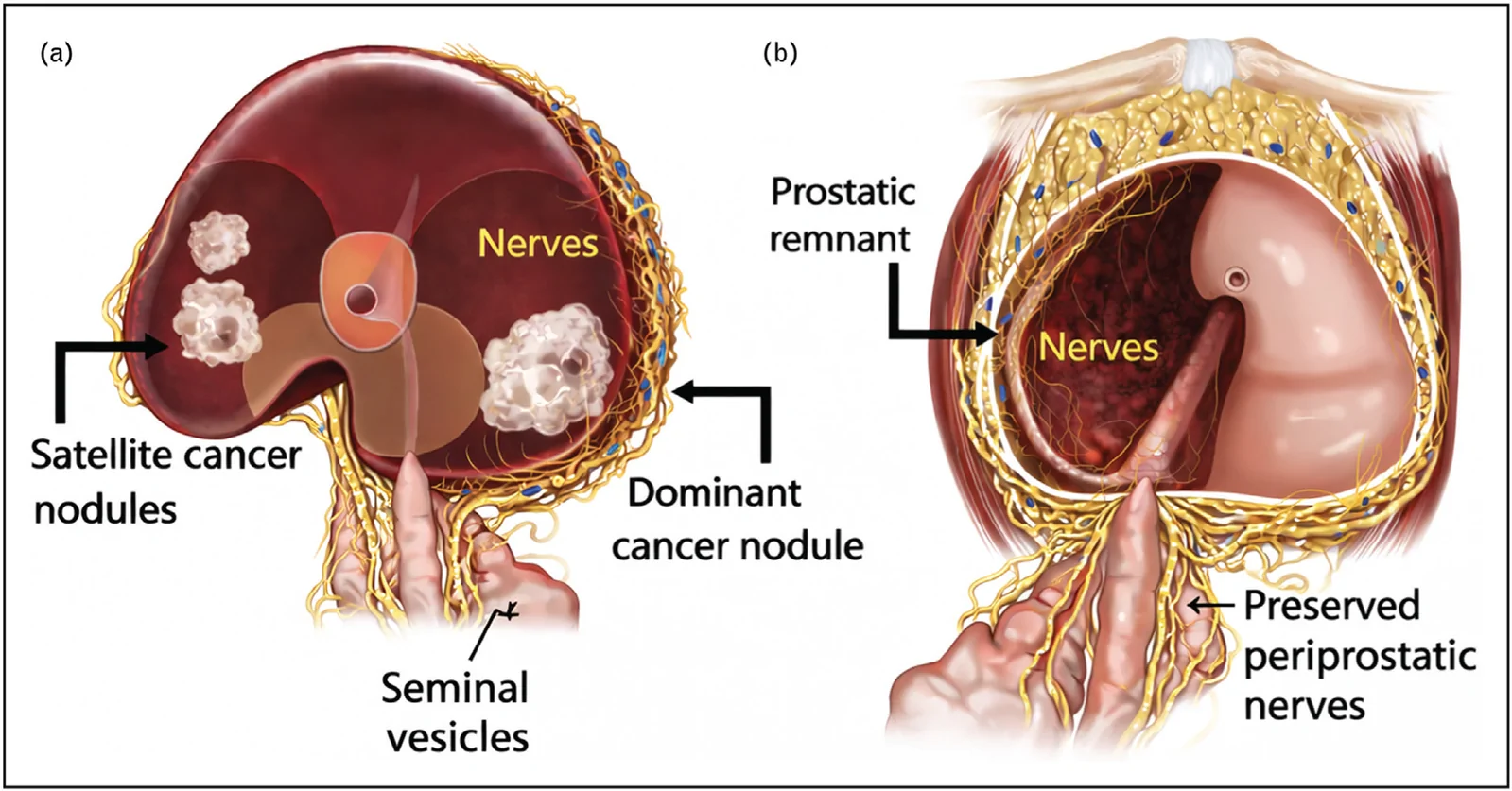
MPP version 1: (a) removed specimen with the precision plane contralateral to the dominant lesion while (b) Remnant contralateral seminal vesicle and capsule with preserved tissue.

The distinction between MPP V1 and standard robotic prostatectomy lies primarily in the nerve-sparing strategy on the side contralateral to the dominant cancer lesion. The surgical technique is highlighted in Fig. 1. On the side of the dominant cancer lesion, nerve sparing is performed according to surgeon's discretion in a standardized risk-adapted approach, which includes the resection of the ipsilateral seminal vesicle complex. On the contralateral side, however, the dissection differs from the conventional plane by a few millimeters. The dissection is initiated anterior to the vas deferens and the seminal vesicles, preserving all layers of the Denonvilliers’ fascia and subsequently proceeds deliberately into the prostatic capsule and superficial parenchyma. A 5–10 mm rim of prostatic tissue is preserved as a protective layer over the neural structures underneath, along with the seminal vesicle complex on the precision site [34].

The underlying concept is the preservation of neural networks beyond the classical neurovascular bundles, with particular attention to the tissue planes between the prostate and the seminal vesicles, which have been shown in anatomical studies to contain a high density of NO-producing nerves [17].

## MPP VERSION 1: OUTCOMES

Following MPP V1, recovery of both erectile and urinary function was rapid in the original IDEAL development cohort [34]. Erectile function represents the principal functional advantage of precision prostatectomy, with patient-reported outcomes demonstrating recovery rates exceeding 90% among preoperatively potent men. At 12 months in the IDEAL stage 1–2b cohort, continence was 100%, pad-free continence was 90.9%, and potency recovery was 90.2% among those potent preoperatively. This observation is further supported by objective measures; prescription renewal of intracavernosal injection (ICI) therapy was significantly lower among patients undergoing MPP V1 when compared to standard radical prostatectomy. When considered in the context of modern nerve-sparing techniques [35], precision prostatectomy demonstrated a favorable pattern of early functional recovery, although randomized comparative data are still lacking. Recent patient-reported outcome data further support this approach. A contemporary analysis of decision regret following precision prostatectomy demonstrated low regret rates at 3% [36^▪^], indicating favorable patient-perceived outcomes when compared to standard prostatectomy cohorts [37].

Given the deliberate preservation of a rim of prostatic tissue, postoperative PSA kinetics may differ from standard radical prostatectomy. In this setting, PSA levels may not reach complete undetectability but rather decline to a nadir; sustained stability at this level is interpreted as indicative of oncologic control. Nevertheless, the overall median postoperative PSA following MPP V1 was reported to be undetectable. At 36 months in the IDEAL stage 1–2b cohort, freedom from clinically significant residual prostate cancer was 93.4%, freedom from secondary treatment was 90.7%, and freedom from biochemical recurrence was 97.2% [34]. A subsequent AUA 2024 abstract reported comparative 5–7-year follow-up for MPP versus standard radical prostatectomy, but these data remain nonrandomized and abstract-only [38].

These outcomes compare favorably with focal therapy cohorts and approach those observed with radical treatment in this selected population. Although the rate of positive surgical margins was somewhat higher, only the presence of Gleason pattern 4 disease was associated with an increased risk of secondary treatment, suggesting that low-grade residual disease may be clinically less relevant in this context. No metastatic events or cancer-specific deaths were observed after undergoing MPP V1 during the reported IDEAL follow-up.

When recurrence is suspected, imaging and remnant biopsy are indicated. Technique-refinement studies have supported the feasibility of transperineal and microultrasound-guided biopsy of the preserved remnant [39]. A small proportion of patients required completion surgery with removal of the preserved remnant for clinically significant residual disease, which was technically straightforward and resulted in undetectable PSA levels without affecting urinary continence. Supporting the role of preserved periprostatic nerve structures in MPP V1, erectile function was consistently lost after salvage resection [34]. Only one patient elected to preserve the seminal vesicle during completion resection of the remnant prostatic capsule. In this patient, no postoperative impairment of erectile function was observed. This observation served as the initial impetus for the development of MPP Version 2.

## MPP VERSION 1: LIMITATIONS

Despite encouraging early oncologic outcomes in the prospective IDEAL cohort, the detection of truly unifocal disease may be a limitation, even in the context of modern multiparametric MRI (mpMRI) and targeted biopsy techniques. Furthermore, the data were generated at high-volume experienced centers. The surgical technique requires advanced expertise in robotic prostate surgery, as plane detection is paramount to perform MPP V1. The technical challenge of preserving a thin rim of prostatic capsule has raised questions about the reproducibility in lower volume settings and the true learning curve of the procedure. MPP V1 is currently under investigation in a randomized controlled trial [40]. However, hesitation among surgeons to deliberately preserve portions of the prostatic capsule has led to the evolution of a modified approach in parallel.

## FUTURE DIRECTIONS: MPP VERSION 2

**FIGURE 2:**
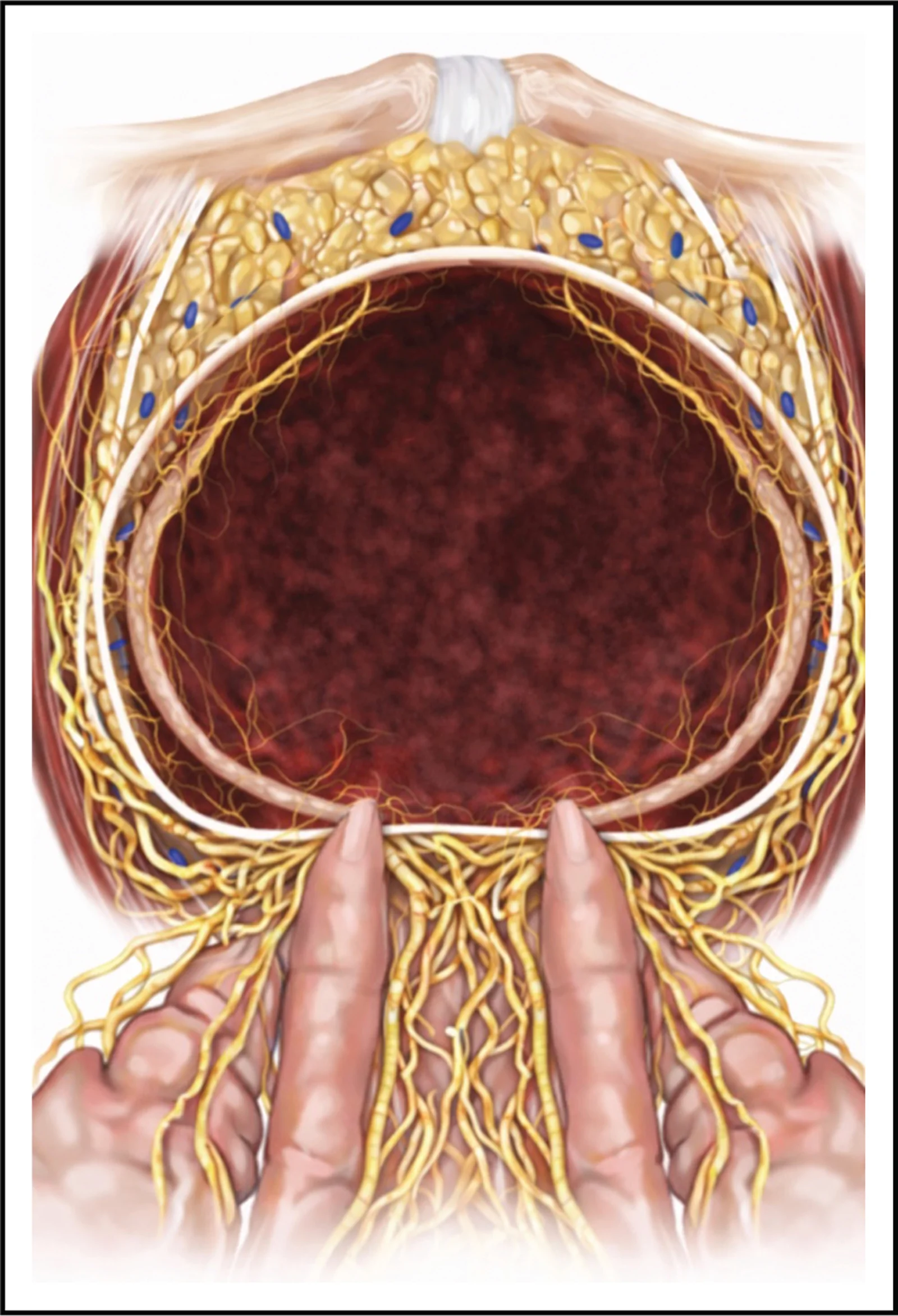
MPP V2 surgical field after MPP V2 with preserved seminal vesicles.

MPP V2 was developed to simplify the surgical approach of precision prostatectomy. The procedure can be conceptualized as a bilateral seminal vesicle-sparing and nerve-sparing RARP with en block resection of the prostate. The underlying rationale is the preservation of periprostatic nerve-rich tissue planes. Specifically, the layers of Denonvilliers’ fascia and the interfaces between the fascia, seminal vesicles, and prostate remain undisturbed, as displayed in Fig. 2.

Previous studies investigated partial preservation of the seminal vesicles, with most reporting functional benefits in terms of urinary continence and erectile function [41–43]; yet, the only randomized controlled trial failed to demonstrate a significant difference [44]. However, all existing approaches are restricted to partial or tip-sparing techniques and do not address preservation of the entire seminal vesicles complex. Consequently, the functional implications of maintaining the full seminal vesicle unit and its surrounding fascial interfaces remain unexplored.

The rationale for MPP V2 is the preservation of the entire seminal vesicle complex rather than only the distal tips. This approach may facilitate retention of basally located periprostatic neural structures enriched in NO synthase–containing nerve fibers, which are potentially relevant to erectile function [17,18]. This approach opens a natural posterior dissection plane, facilitating an intrafascial nerve dissection with preservation of the lateral veil of nerve tissue that can also be continued anteriorly to preserve anterior tissues, similar to a hood technique [23,25^▪^,^35^].

## PATIENT SELECTION AND FUTURE DIRECTION OF MPP V2

MPP V2 remains under ongoing investigation. As with its preceding iteration, patient selection is key. Patients offered MPP V2 should place a high priority on maintaining their erectile function. Its oncologic safety is largely determined by the ability to reliably exclude seminal vesicle invasion (SVI). Despite advances in imaging, preoperative detection remains imperfect, and negative imaging cannot reliably exclude microscopic involvement [45]. Anatomically, SVI originates at the prostatic base, either through direct spread along the ejaculatory duct sheath or via capsular penetration [46].

Established nomograms identify PSA, ISUP grade, and tumor burden as key predictors of SVI; however, most fail to account for tumor location [47–53]. Tumors involving the prostatic base may carry a substantially higher risk, whereas absence of base involvement may identify patients at very low risk. The Koh nomogram uniquely incorporates the location and extent of cancer in base biopsy cores. The clinical relevance of this is underscored by findings from the original series: among 275 patients with PSA 10 ng/ml or less and no cancer detected in base biopsy cores, the rate of SVI at final pathology was 0% [49].

When applied to MPP V2, these findings suggest that incorporation of tumor location may refine patient selection. Preliminary criteria may include PSA less than 10 ng/ml, ISUP grade 2 disease with limited tumor burden, PI-RADS 4 or less, and exclusion of tumors involving the prostatic base. These criteria should be considered hypothesis-generating and require prospective validation.

Additional oncologic safety may be supported by intraoperative adjuncts such as frozen section analysis or emerging technologies such as NeuroSAFE [54,55] or Histolog confocal microscopy [56,57]. Advanced imaging approaches, including PSMA-targeted fluorescence-guided surgery [58–60] and artificial intelligence-assisted image integration [61–63] may further enhance intraoperative tumor localization in the future. Telementoring may facilitate real-time expert guidance across institutions [64–67].

In terms of oncologic follow-up, the existing evidence of seminal vesicle-tip-sparing surgery suggests that even though the residual seminal vesicle may produce low levels of PSA when left *in situ*, this is typically only detectable on ultrasensitive assays, and importantly, did not result in worse biochemical recurrence rates compared to standard surgery [68].

MPP remains under clinical investigation [40] and may represent a surgical option for carefully selected patients with favorable intermediate-risk disease and a strong preference for preservation of erectile function, ideally at a high-volume center within a clinical trial.

## CONCLUSION

Persistent functional morbidity following radical prostatectomy and emerging anatomical insights into the complexity of periprostatic innervation, has driven the development of precision prostatectomy as surgical strategy to prevent postoperative erectile dysfunction. MPP V1 demonstrated that preservation of selected prostatic and periprostatic tissue planes can translate into rapid and superior functional recovery while maintaining encouraging oncologic outcomes. However, the technical demands of the procedure and the reliance on highly specialized centers raise concerns regarding reproducibility and generalizability. MPP V2 represents an evolution of this concept, aiming to simplify the surgical approach while further preserving nerve-rich anatomical planes, particularly through bilateral preservation of the seminal vesicle complex. Ongoing investigation will determine whether this refined technique can safely extend the principles of surgical de-escalation by improving functional outcomes for patients undergoing prostatectomy.

## Acknowledgements

*None*.

*Author contributions J.F. contributed to conceptualization, literature review, and drafting of the manuscript. C.R. contributed to conceptualization and critical revision of the manuscript. L.Z. contributed to critical revision of the manuscript. K.B. contributed to critical revision of the manuscript. M.M. contributed to conceptualization, senior supervision, and critical revision of the manuscript.*

### Financial support and sponsorship

*None.*

### Conflicts of interest

*There are no conflicts of interest.*

*Generative AI: Generative AI was used for minor proofreading and to modify figures for which the last author holds the copyright. The conceptualization, drafting, and substantive writing of the manuscript were performed by the authors without the use of generative AI.*

## REFERENCES AND RECOMMENDED READING

Papers of particular interest, published within the annual period of review, have been highlighted as:

▪ of special interest
▪▪ of outstanding interest

## References

[1] Meissner VH Woll M Ankerst DP. Long-term and pathological outcomes of low- and intermediate-risk prostate cancer after radical prostatectomy: implications for active surveillance. World J Urol 2021; 39:3763–3770. 33973043 10.1007/s00345-021-03717-2 PMC8521579

[2] Wilt TJ Brawer MK Jones KM Prostate Cancer Intervention versus Observation Trial (PIVOT) Study Group. Radical prostatectomy versus observation for localized prostate cancer. N Engl J Med 2012; 367:203–213. 22808955 10.1056/NEJMoa1113162 PMC3429335

[3] Bill-Axelson A Holmberg L Garmo H. Radical prostatectomy or watchful waiting in prostate cancer — 29-year follow-up. N Engl J Med 2018; 379:2319–2329. 30575473 10.1056/NEJMoa1807801

[4] Bill-Axelson A Holmberg L Garmo H. Radical prostatectomy or watchful waiting in early prostate cancer. N Engl J Med 2014; 370:932–942. 24597866 10.1056/NEJMoa1311593 PMC4118145

[5] Hamdy FC Donovan JL Lane JA ProtecT Study Group. 10-year outcomes after monitoring, surgery, or radiotherapy for localized prostate cancer. N Engl J Med 2016; 375:1415–1424. 27626136 10.1056/NEJMoa1606220

[6] Nepple KG Stephenson AJ Kallogjeri D. Mortality after prostate cancer treatment with radical prostatectomy, external-beam radiation therapy, or brachytherapy in men without comorbidity. Eur Urol 2013; 64:372–378. 23506834 10.1016/j.eururo.2013.03.005 PMC3930076

[7] Ficarra V Novara G Ahlering TE. Systematic review and meta-analysis of studies reporting potency rates after robot-assisted radical prostatectomy. Eur Urol 2012; 62:418–430. 22749850 10.1016/j.eururo.2012.05.046

[8▪▪] Dinneen E Almeida-Magana R Al-Hammouri T NeuroSAFE PROOF Investigators. Effect of NeuroSAFE-guided RARP versus standard RARP on erectile function and urinary continence in patients with localised prostate cancer (NeuroSAFE PROOF): a multicentre, patient-blinded, randomised, controlled phase 3 trial. Lancet Oncol 2025; 26:447–458. 40147459 10.1016/S1470-2045(25)00091-9

[9▪] Ślusarczyk A Gurwin A Barnaś A Section of Urologic Oncology of Polish Urological Association. Outcomes of focal therapy for localized prostate cancer: a systematic review and meta-analysis of prospective studies. Eur Urol Oncol 2025; 8:1653–1672. 40251100 10.1016/j.euo.2025.02.003

[10] Blank F Meyer M Wang H. Salvage radical prostatectomy after primary focal ablative therapy: a systematic review and meta-analysis. Cancers (Basel) 2023; 15:2727. 37345064 10.3390/cancers15102727 PMC10216462

[11] Finkle AL Saunders JB de CM. Sexual potency in aging males. III. Technic of avoiding nerve injury in perineal prostatic operations. Am J Surg 1960; 99:23–26. 13822916 10.1016/0002-9610(60)90243-9

[12] Walsh PC. The discovery of the cavernous nerves and development of nerve sparing radical retropubic prostatectomy. J Urol 2007; 177:1632–1635. 17437775 10.1016/j.juro.2007.01.012

[13] Walsh PC Donker PJ. Impotence following radical prostatectomy: insight into etiology and prevention. J Urol 1982; 128:492–497. 7120554 10.1016/s0022-5347(17)53012-8

[14] Takenaka A Tewari A Hara R. Pelvic autonomic nerve mapping around the prostate by intraoperative electrical stimulation with simultaneous measurement of intracavernous and intraurethral pressure. J Urol 2007; 177:225–229. 17162051 10.1016/j.juro.2006.08.104

[15] Sievert KD Hennenlotter J Laible I. The periprostatic autonomic nerves—bundle or layer? Eur Urol 2008; 54:1109–1117. 18621470 10.1016/j.eururo.2008.06.007

[16] Ganzer R Stolzenburg JU Wieland WF Bründl J. Anatomic study of periprostatic nerve distribution: immunohistochemical differentiation of parasympathetic and sympathetic nerve fibres. Eur Urol 2012; 62:1150–1156. 22469393 10.1016/j.eururo.2012.03.039

[17] Ganzer R Stolzenburg JU Neuhaus J. Anatomical study of pelvic nerves in relation to seminal vesicles, prostate and urethral sphincter: immunohistochemical staining, computerized planimetry and 3-dimensional reconstruction. J Urol 2015; 193:1205–1212. 25301095 10.1016/j.juro.2014.10.001

[18] Yang SY Kim HS Cho MS Kim NK. Three-dimensional anatomy of the Denonvilliers’ fascia after micro-CT reconstruction. Sci Rep 2021; 11:21759. 34741081 10.1038/s41598-021-01106-8 PMC8571354

[19] Hernández V Espinos EL Dunn J. Oncological and functional outcomes of sexual function-preserving cystectomy compared with standard radical cystectomy in men: a systematic review. Urologic oncology 2017; 35:539.e17–539.e29. 10.1016/j.urolonc.2017.04.013 28495555

[20] Shumaker L Leopold Z Perilstein M. Robot-assisted transvesical simple prostatectomy with circumferential mucosal anastomosis: long-term urinary and sexual function outcomes in a 292 patient cohort. J Robot Surg 2025; 19:693–1693. 41099878 10.1007/s11701-025-02879-0 PMC12531315

[21] Krishnan R Katz D Nelson CJ Mulhall JP. Erectile function recovery in patients after nonnerve sparing radical prostatectomy. Andrology 2014; 2:951–954. 25270277 10.1111/andr.282

[22] Kim NK Aahn TW Park JK. Assessment of sexual and voiding function after total mesorectal excision with pelvic autonomic nerve preservation in males with rectal cancer. Dis Colon Rectum 2002; 45:1178–1185. 12352233 10.1007/s10350-004-6388-5

[23] Menon M Kaul S Bhandari A. Potency following robotic radical prostatectomy: a questionnaire based analysis of outcomes after conventional nerve sparing and prostatic fascia sparing techniques. Journal of Urology 2005; 174:2291–2296. 16280816 10.1097/01.ju.0000181825.54480.eb

[24] Tewari AK Srivastava A Huang MW. Anatomical grades of nerve sparing: a risk-stratified approach to neural-hammock sparing during robot-assisted radical prostatectomy (RARP). BJU Int 2011; 108 (6 Pt 2):984–992. 21917101 10.1111/j.1464-410X.2011.10565.x

[25▪] Mandel A Choudhary M Tillu N. Hood technique for radical prostatectomy. J Endourol 2025; 39 (S1):S35–S38. 40100831 10.1089/end.2024.0303

[26] Liu W Laitinen S Khan S. Copy number analysis indicates monoclonal origin of lethal metastatic prostate cancer. Nat Med 2009; 15:559–565. 19363497 10.1038/nm.1944 PMC2839160

[27] Woodcock DJ Riabchenko E Taavitsainen S. Prostate cancer evolution from multilineage primary to single lineage metastases with implications for liquid biopsy. Nat Commun 2020; 11:5070. 33033260 10.1038/s41467-020-18843-5 PMC7545111

[28] Ahmed HU Hindley RG Dickinson L. Focal therapy for localised unifocal and multifocal prostate cancer: a prospective development study. Lancet Oncol 2012; 13:622–632. 22512844 10.1016/S1470-2045(12)70121-3 PMC3366323

[29] Golan R Bernstein AN McClure TD. Partial gland treatment of prostate cancer using high-intensity focused ultrasound in the primary and salvage settings: a systematic review. J Urol 2017; 198:1000–1009. 28433640 10.1016/j.juro.2017.03.137

[30] Bass R Fleshner N Finelli A. Oncologic and functional outcomes of partial gland ablation with high intensity focused ultrasound for localized prostate cancer. J Urol 2019; 201:113–119. 10.1016/j.juro.2018.07.040 30036514

[31] Kenigsberg AP Llukani E Deng FM. The use of magnetic resonance imaging to predict oncological control among candidates for focal ablation of prostate cancer. Urology 2018; 112:121–125. 29061480 10.1016/j.urology.2017.10.014

[32] Shah TT Peters M Eldred-Evans D. Early-medium-term outcomes of primary focal cryotherapy to treat nonmetastatic clinically significant prostate cancer from a prospective multicentre registry. Eur Urol 2019; 76:98–105. 30638633 10.1016/j.eururo.2018.12.030

[33] Sood A Jeong W Taneja K. The precision prostatectomy: an IDEAL Stage 0, 1 and 2a Study. BMJ Surg Interv Health Technol 2019; 1:e000002. 10.1136/bmjsit-2019-000002 PMC8647607 35047770

[34] Sood A Jeong W Palma-Zamora I. Description of surgical technique and oncologic and functional outcomes of the precision prostatectomy procedure (IDEAL Stage 1-2b Study). Eur Urol 2022; 81:396–406. 34872786 10.1016/j.eururo.2021.10.017

[35] Tewari AK Srivastava A Huang MW. Anatomical grades of nerve sparing: a risk-stratified approach to neural-hammock sparing during robot-assisted radical prostatectomy (RARP). BJU International 2011; 108 (6 pt 2):984–992. 21917101 10.1111/j.1464-410X.2011.10565.x

[36▪] Kolanukuduru KP Jeong W Pistin L. Treatment decision regret after precision prostatectomy: an analysis of patient-reported outcomes predicting decision regret. BJUI Compass 2025; 6:e476. 39877582 10.1002/bco2.476 PMC11771488

[37] Schroeck FR Krupski TL Sun L. Satisfaction and regret after open retropubic or robot-assisted laparoscopic radical prostatectomy. Eur Urol 2008; 54:785–793. 18585849 10.1016/j.eururo.2008.06.063

[38] Kolanukuduru KP Tinsley S Sood A. MP52–16 comparative outcomes of standard and modified radical prostatectomy at 5–7 years of follow-up. J Urol 2026; 211:e859.

[39] Grauer R Gorin MA Sood A. Impact of prostate biopsy technique on outcomes of the precision prostatectomy procedure. BMJ Surg Interv Health Technol 2022; 4:e000122. 10.1136/bmjsit-2021-000122 PMC9260793 35892060

[40] Abdollah F. A randomized comparison of radical prostatectomy and precision prostatectomy in low- and intermediate-risk prostate cancers. clinicaltrials.gov; 2026 Jan [cited 26 April 2026]. Clinical trial registration no.: NCT07348367. Available at: https://clinicaltrials.gov/study/NCT07348367.

[41] John H Hauri D. Seminal vesicle-sparing radical prostatectomy: a novel concept to restore early urinary continence. Urology 2000; 55:820–824. 10840084 10.1016/s0090-4295(00)00547-1

[42] Bellina M Mari M Ambu A. Seminal monolateral nerve-sparing radical prostatectomy in selected patients. Urol Int 2005; 75:175–180. 16123574 10.1159/000087174

[43] Albers P Schäfers S Löhmer H de Geeter P. Seminal vesicle-sparing perineal radical prostatectomy improves early functional results in patients with low-risk prostate cancer. BJU Int 2007; 100:1050–1054. 17760889 10.1111/j.1464-410X.2007.07123.x

[44] Gilbert SM Dunn RL Miller DC. Functional outcomes following nerve sparing prostatectomy augmented with seminal vesicle sparing compared to standard nerve sparing prostatectomy: results from a randomized controlled trial. J Urol 2017; 198:600–607. 28392393 10.1016/j.juro.2017.03.133

[45] Woo S Ghafoor S Becker AS. Prostate-specific membrane antigen positron emission tomography (PSMA-PET) for local staging of prostate cancer: a systematic review and meta-analysis. Eur J Hybrid Imaging 2020; 4:16. 34191215 10.1186/s41824-020-00085-9 PMC8218057

[46] Villers AA McNeal JE Redwine EA. Pathogenesis and biological significance of seminal vesicle invasion in prostatic adenocarcinoma. J Urol 1990; 143:1183–1187. 2342179 10.1016/s0022-5347(17)40220-5

[47] Martini A Gupta A Cumarasamy S. Novel nomogram for the prediction of seminal vesicle invasion including multiparametric magnetic resonance imaging. Int J Urol 2019; 26:458–464. 30659663 10.1111/iju.13905

[48] Kattan MW Wheeler TM Scardino PT. Postoperative nomogram for disease recurrence after radical prostatectomy for prostate cancer. J Clin Oncol 1999; 17:1499–1507. 10334537 10.1200/JCO.1999.17.5.1499

[49] Koh H Kattan MW Scardino PT. A nomogram to predict seminal vesicle invasion by the extent and location of cancer in systematic biopsy results. J Urol 2003; 170 (4 Pt 1):1203–1208. 14501725 10.1097/01.ju.0000085074.62960.7b

[50] Gallina A Chun FKH Briganti A. Development and split-sample validation of a nomogram predicting the probability of seminal vesicle invasion at radical prostatectomy. Eur Urol 2007; 52:98–105. 17267098 10.1016/j.eururo.2007.01.060

[51] Gandaglia G Ploussard G Valerio M. The key combined value of multiparametric magnetic resonance imaging, and magnetic resonance imaging-targeted and concomitant systematic biopsies for the prediction of adverse pathological features in prostate cancer patients undergoing radical prostatectomy. Eur Urol 2020; 77:733–741. 31547938 10.1016/j.eururo.2019.09.005

[52] Hall WA Fishbane N Liu Y. Development and validation of a genomic tool to predict seminal vesicle invasion in adenocarcinoma of the prostate. JCO Precis Oncol 2020; 4:1228–1238. 35050780 10.1200/PO.20.00013

[53] Tosoian JJ Chappidi M Feng Z. Prediction of pathological stage based on clinical stage, serum prostate-specific antigen, and biopsy Gleason score: Partin Tables in the contemporary era. BJU Int 2017; 119:676–683. 27367645 10.1111/bju.13573

[54] Dinneen E Almeida-Magana R Al-Hammouri T NeuroSAFE PROOF Investigators. Effect of NeuroSAFE-guided RARP versus standard RARP on erectile function and urinary continence in patients with localised prostate cancer (NeuroSAFE PROOF): a multicentre, patient-blinded, randomised, controlled phase 3 trial. Lancet Oncol 2025; 26:447–458. 40147459 10.1016/S1470-2045(25)00091-9

[55] Kroon LJ Van Der Slot MA Van Den Bergh RCN. Neurovascular Structure-adjacent Frozen-section Examination (NeuroSAFE) during radical prostatectomy: a systematic review and meta-analysis. Eur Urol Oncol 2025; 8:1365–1374. 39730246 10.1016/j.euo.2024.12.008

[56] Almeida-Magana R Au M Al-Hammouri T. Accuracy of the LaserSAFE technique for detecting positive surgical margins during robot-assisted radical prostatectomy: blind assessment and inter-rater agreement analysis. Histopathology 2025; 86:433–440. 39403832 10.1111/his.15336 PMC11707496

[57] Tewari AK. Feasibility of timesaving when using histolog confocal laser endomicroscopy for margin assessment in prostatectomy specimen [Clinical trial registration] [Internet]. clinicaltrials.gov; 2025 Oct [cited 26 April 2026]. Clinical trial registration no.: NCT07141121. Available at: https://clinicaltrials.gov/study/NCT07141121.

[58] Stibbe JA de Barros HA Linders DGJ. First-in-patient study of OTL78 for intraoperative fluorescence imaging of prostate-specific membrane antigen-positive prostate cancer: a single-arm, phase 2a, feasibility trial. Lancet Oncol 2023; 24:457–467. 37062295 10.1016/S1470-2045(23)00102-X

[59] Li P Xu Y Yu S. First-in-human study of DGPR1008 for intraoperative fluorescence imaging of prostate-specific membrane antigen-positive prostate cancer in patients undergoing radical prostatectomy. Transl Androl Urol 2025; 14:2697–2709. 41132338 10.21037/tau-2025-537 PMC12541503

[60] Bahler C. Phase II Trial to Investigate the Safety and Efficacy of Four Dosing Regimens of OTL78 Injection (Zopocianine), a Prostate-Specific Membrane Antigen (PSMA)-Targeted Fluorescent Agent, for the Intraoperative Imaging of Prostate Cancer [Clinical trial registration] [Internet]. clinicaltrials.gov; 2026 Feb [cited 26 April 2026]. Clinical trial registration no.: NCT06849544. Available from: https://clinicaltrials.gov/study/NCT06849544.

[61] Porpiglia F Fiori C Checcucci E. Augmented reality robot-assisted radical prostatectomy: preliminary experience. Urology 2018; 115:184. 29548868 10.1016/j.urology.2018.01.028

[62] Checcucci E Piana A Volpi G. Three-dimensional automatic artificial intelligence driven augmented-reality selective biopsy during nerve-sparing robot-assisted radical prostatectomy: a feasibility and accuracy study. Asian J Urol 2023; 10:407–415. 38024433 10.1016/j.ajur.2023.08.001 PMC10659972

[63] Carbin DD Shah A Kusuma VRM. Artificial intelligence in robot-assisted radical prostatectomy: where do we stand today? J Robotic Surg 2024; 18:404. 10.1007/s11701-024-02143-x 39527349

[64] Yamamoto A Morizane S Shimizu R. Initial experience of telementoring using the intuitive telepresence® systems for robot-assisted radical prostatectomy. J Endourol 2026; 40:313–320. 41810590 10.1177/08927790261416871

[65] Shin DH Dalag L Azhar RA. A novel interface for the telementoring of robotic surgery. BJU Int 2015; 116:302–308. 25381917 10.1111/bju.12985 PMC9083498

[66] Faris H Harfouche C Bandle J Wisbach G. Surgical tele-mentoring using a robotic platform: initial experience in a military institution. Surg Endosc 2023; 37:9159–9166. 37821559 10.1007/s00464-023-10484-1 PMC10709226

[67] Erridge S Yeung DKT Patel HRH Purkayastha S. Telementoring of surgeons: a systematic review. Surg Innov 2019; 26:95–111. 30465477 10.1177/1553350618813250

[68] Burkhardt O Neuenschwander J John H Randazzo M. Does seminal vesicle-sparing robotic radical prostatectomy influence postoperative prostate-specific antigen measured with an ultrasensitive immunoassay? Swiss Med Wkly 2018; 148:w14685–w114685. 30474852 10.4414/smw.2018.14685

